# Treatment and outcome of giant cell tumors of the pelvis: 20 cases followed for 1 to 11 years

**DOI:** 10.3109/17453670903350123

**Published:** 2009-10-01

**Authors:** Maurice Balke, Arne Streitbuerger, Tymoteusz Budny, Marcel Henrichs, Georg Gosheger, Jendrik Hardes

**Affiliations:** Department of Orthopedic Surgery, University Hospital MuensterMuensterGermany

## Abstract

**Background and purpose** Giant cell tumors (GCTs) of bone rarely affect the pelvis. We report on 20 cases that have been treated at our institution during the last 20 years.

**Methods** 20 patients with histologically benign GCT of the pelvis were included in this study. 9 tumors were primarily located in the iliosacral area, 6 in the acetabular area, and 5 in the ischiopubic area. 8 patients were treated by intralesional curettage and 6 by intralesional resection with additional curettage of the margins. 3 patients with iliacal tumors were treated by wide resection. 2 patients were treated by a combination of external beam irradiation and surgery, and 1 patient solely by irradiation. In addition, 9 patients received selective arterial embolization one day before surgery. Of the 6 patients with acetabular tumors, 1 secondarily received an endoprosthesis and 1 was primarily treated by hip transposition. The patients were followed for a median time of 3 (1–11) years.

**Results** 1 patient with a pubic tumor developed a local recurrence 1 year after intralesional resection and additional curettage of the margins. The recurrence presented as a small soft tissue mass within the scar tissue of the gluteal muscles and was treated by resection. No secondary sarcoma was detected and none of the patients developed pulmonary metastases or multicentricity. No major complication occurred during surgery.

**Interpretation** We conclude that most GCTs of the pelvis can be treated by intralesional procedures. For tumors of the iliac wing, wide resection can be an alternative. Surgical treatment of tumors affecting the acetabular region often results in functional impairment. Pre-surgical selective arterial embolization appears to be a safe procedure that may reduce the risk of local recurrence.

## Introduction

The most commonly recommended treatment for giant cell tumors (GCTs) of bone consists of intralesional procedures such as curettage, and filling of the defect either with bone grafts or bone cement. The local recurrence rate depends on the surgical treatment and can be up to 40% (Persson et al. 1984, Campanacci et al. 1987, Blackley et al. 1999, Malek et al. 2006, Kivioja et al. 2008). A recent study has shown that the lowest rates in local recurrence (approximately 10%) are achieved by intralesional curettage followed by a combination of different adjuncts such as high-speed burring, lavage with hydrogen peroxide (H_2_O_2_), and bone cement packing (polymethyl methacrylate; PMMA) (Balke et al. 2008b). Although the recurrence rates are even lower with wide resection, this procedure seems to be an overtreatment and is usually not recommended (Balke and Schremper et al. 2008, Kivioja et al. 2008).

These findings apply to “typical” tumors that occur in the long bones, but occasionally GCTs also arise in the vertebrae, pelvis, and sacrum (Campanacci et al. 1987, Osaka and Toriyama 1987, Shikata et al. 1992, Sanjay et al. 1993, Leggon et al. 2004, Balke et al. 2008b). Optimal treatment for these locations is still controversial. The tumors may reach remarkable sizes, especially in the pelvis. Due to anatomical difficulties such as potential impairment of iliac vessels, the bony support of the pelvic ring, the hip joint, and intra-abdominal organs, surgery may be challenging and risky. Thus, alternative treatments such as irradiation are taken into account although the recurrence rate without surgical removal is high. Moreover, there is the risk of radiation-induced secondary sarcoma (Schwartz et al. 1989, Chakravarti et al. 1999, Leggon et al. 2004). Very few publications have specifically addressed GCT of the pelvis. To our knowledge, only 3 papers on this topic are available in the literature in English (Osaka and Toriyama 1987, Sanjay et al. 1993, Leggon et al. 2004). The largest series, with 19 cases treated within a 46-year period, was published by Sanjay et al. (1993).

Here we report on another 20 cases that have been treated at a single institution during the past 20 years. We lay special emphasis on different treatment modalities with respect to occurrence of local recurrence, pulmonary metastases, functional outcome, and complications.

## Methods

243 patients with histologically benign GCT of bone were treated at the authors' institution from 1980 to 2008. 20 of these patients (12 women) who presented with GCT of the pelvis are included in this study. 16 patients had received the initial treatment at our institution and 4 patients had been referred because of estimated recurrence or due to complications (Table).

**Table:** Data on 20 patients with giant cell tumor of bone

A	B	C	D	E	F	G	H	I	J	K	L	M	N
1	M	38	I	Ilium with sacral extension	L	3	+	A	SAE, curettage, burring + PMMA 03/99	–	19	NED	
2	M	33	I	Ilium with sacral extension	L	3	+	A	SAE, curettage, H_2_O_2_, cryotherapy + PMMA 06/95	–	38	NED	+
3	F	44	I	Ilium	L	3	+	A	Curettage + PMMA 12/89	–	30	NED	+
4	F	17	I	Ilium	L	n.d.		A	Curettage + bone graft 01/90	–	34	NED	+
5	F	35	I	Ilium with sacral extension	L	3	+	B	SAE, curettage + PMMA, partial sacral resection 09/89	–	128	NED	
6	F	35	I	Ilium	R	3	+	C	SAE, wide resection of ilium 11/04	–	33	NED	
7	M	65	I	Ilium	L	2	+	C	Wide resection of ilium 06/05	–	46	NED	+
8	M	27	I	Ilium	R	3	+	C	Wide resection of ilium 10/04	–	55	NED	+
9	F	35	I	Ilium with sacral extension and extension to lumbar spine (L5)	R	3	nd	D	EBI with 50 Gy 05/97–07/97, SAE, ILR + curettage of margins, H_2_O_2_ + PMMA 09/97	–	123	NED	+
10	F	57	III–II	Ischiopubis with acetabular extension and path. fracture	R	3	–	A	SAE, curettage, burring + PMMA 10/00	–	87	NED	+
11	F	43	III–II	Ischium with acetabular extension	R	3	+	B	SAE, ILR + curettage of margins + PMMA 09/05	–	45	NED	+
12	F	29	III–II	Pubis with acetabular extension	R	3	+	B	SAE, ILR + curettage of margins + hip transposition 02/00	–	112	NED	+
13	M	31	III–II	Ischium with acetabular extension	L	3	+	B	SAE, ILR + curettage and burring of margins, PMMA 05/05	–	22	NED	+
14	F	60	III–II	Pubis with acetabular extension	L	3	+	D	Curettage + cryotherapy + bone graft 10/95, adjuvant EBI with 50 Gy 01/96–03/96	–	20	NED	
15	M	47	III–II	Ischiopubis with acetabular extension and soft tissue infiltration of adductor muscles	L	3	+	E	Sole treatment with EBI using proton beam radiation 10/04–12/04	–	30	SD	+
16	F	38	III	Ischium	L	3	+	A	Curettage, burring and H_2_O_2_ + bone graft + PMMA 07/00	–	14	NED	
17	F	48	III	Pubis	L	n.d.		A	Curettage, phenol + bone graft 01/99	–	13	NED	+
18	M	52	III	Pubis	L	2	+	A	Curettage, burring + PMMA 08/01	–	72	NED	
19	F	54	III	Pubis	L	2	–	B	ILR + curettage and burring of margins 04/07	+	25	NED	+
20	M	59	III	Pubis	R	3	–	B	ILR + curettage and burring of margins + PMMA 02/08	–	12	NED	
A Case														B Sex														C Age														D Region / site of lesion: Grouping of anatomical site (Enneking and Dunham 1978, Sanjay et al. 1993): I, iliosacral; II, ischiopubic with acetabular extension; III, ischiopubic.														E Region / site of lesion														F Side: L, left; R, right.														G Stage														H Soft tissue extension: –, no; +, yes; n.d., not documented.														I Grouping of treatment regimen: A, intralesional curettage; B, intralesional resection of mainly affected bone and extension of margins by curettage; C, wide resection; D, external beam irradiation (EBI) combined with surgery; E, EBI with proton beam radiation.														J Treatment: PMMA, polymethyl methacrylate (bone cement); ILR, intralesional resection; SAE, selective arterial embolization; EBI, external beam irradiation.														K Local recurrence: –, no; +, yes.														L Follow-up (months).														M Status: NED, no evidence of disease; SD, stable disease; ABC: aneurysmal bone cyst.														N Comments: +, yes. See below. Case:														2. Secondary aneurysmal bone cyst.				3. Referred to us due to local infection after biopsy in another hospital; 3 revisions in 01/99.	4. Treatment in other hospital, referred to us to exclude recurrence in 10/92.	7. Limping using a cane.	8. Secondary aneurysmal bone cyst.	9. Referred to us 4 months after biopsy and progressive disease. Postoperative impaired wound healing and local infection, resection, and hip transposition 10/97, removal of PMMA + debridement 11/97.	10. Endoprosthesis with acetabular reconstruction in 06/01 due to femoral head necrosis; limping, using 1 cane for longer walks.	11. Dislocation of screw for PMMA fixation, local pain, removal of screw in 01/09.	12. Secondary ABC, resection of scar tissue in 04/01, subluxation of femoral head; limping but free from pain; use of cane for longer walks.	13. Dislocation of screw for PMMA fixation in 08/05, removal of screw; occasionally minimal pain.	15. Stable disease, additional treatment with oral bisphosphonates, acetabular protrusion.	17. Treatment in other hospital, referred to us to exclude recurrence in 09/99.	19. Secondary ABC, soft tissue recurrence treated by wide resection in 04/08; occasionally moderate pain in pubic and gluteal area while sitting.	20. Surgical excision of sigmoidal gastrointestinal stroma tumor in 01/08; occasionally minimal local pain.

The data was retrospectively collected based on information from patient records, surgical protocols, and histological and radiological findings. The last follow-up was done via personal or telephone contact.

Most patients were aged between the fourth and sixth decade of life at first diagnosis, with a median age of 41 (17–65) years. The median follow-up period was 3 (1–11) years.

### Localization

**Figure 1.**
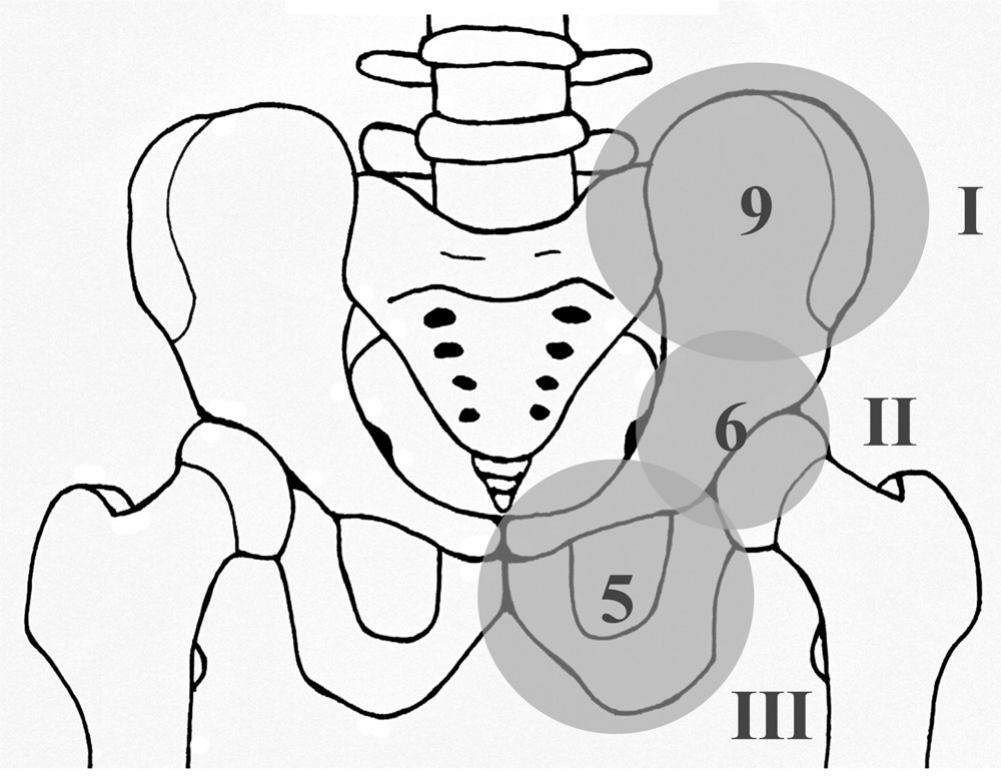
Localization of main tumor components. 3 groups according to Enneking and Dunham (1978). Region I: iliosacral area; region II: acetabular area; region III: ischiopubic area. 9 tumors affected region I, 6 affected region II, and 5 affected region III.

According to the classification system by Enneking and Dunham (1978) for pelvic tumors, as modified by Sanjay et al. (1993), the localizations were divided into 3 groups (Figure 1): 9 tumors were located in region I (iliosacral), 4 of which also showed sacral extension, 5 tumors solely affected region III (ischiopubic), and 6 tumors involved the acetabular region—referred to as region II.

Concerning the radiological findings, 15 patients presented with a stage III lesion, 3 patients presented with a stage II lesion, and none of the patients presented with a stage I lesion according to Campanacci et al. (1987). The radiographs of 2 patients were no longer available.

### Treatment

**Figure 2.**
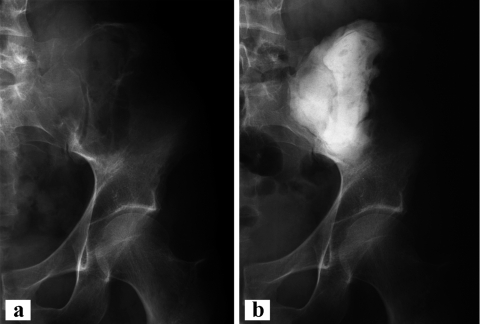
Case 2. a. GCT of the left ilium with sacral extension and proximal cortical breakthrough. b. After intralesional curettage and bone cement packing of the defect.

**Figure 3.**
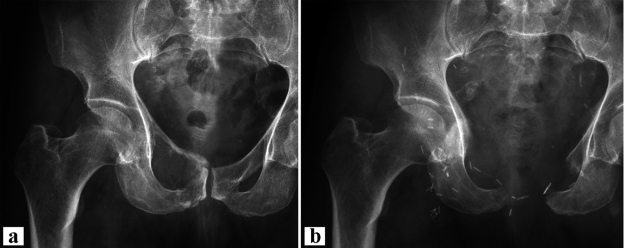
Case 20. a. GCT of the right pubic bone with bony destruction. b. After intralesional resection of pubic bone, including the symphysis. Proximal margins were additionally curetted and cemented. Note the multiple hemostatic clips.

**Figure 4.**
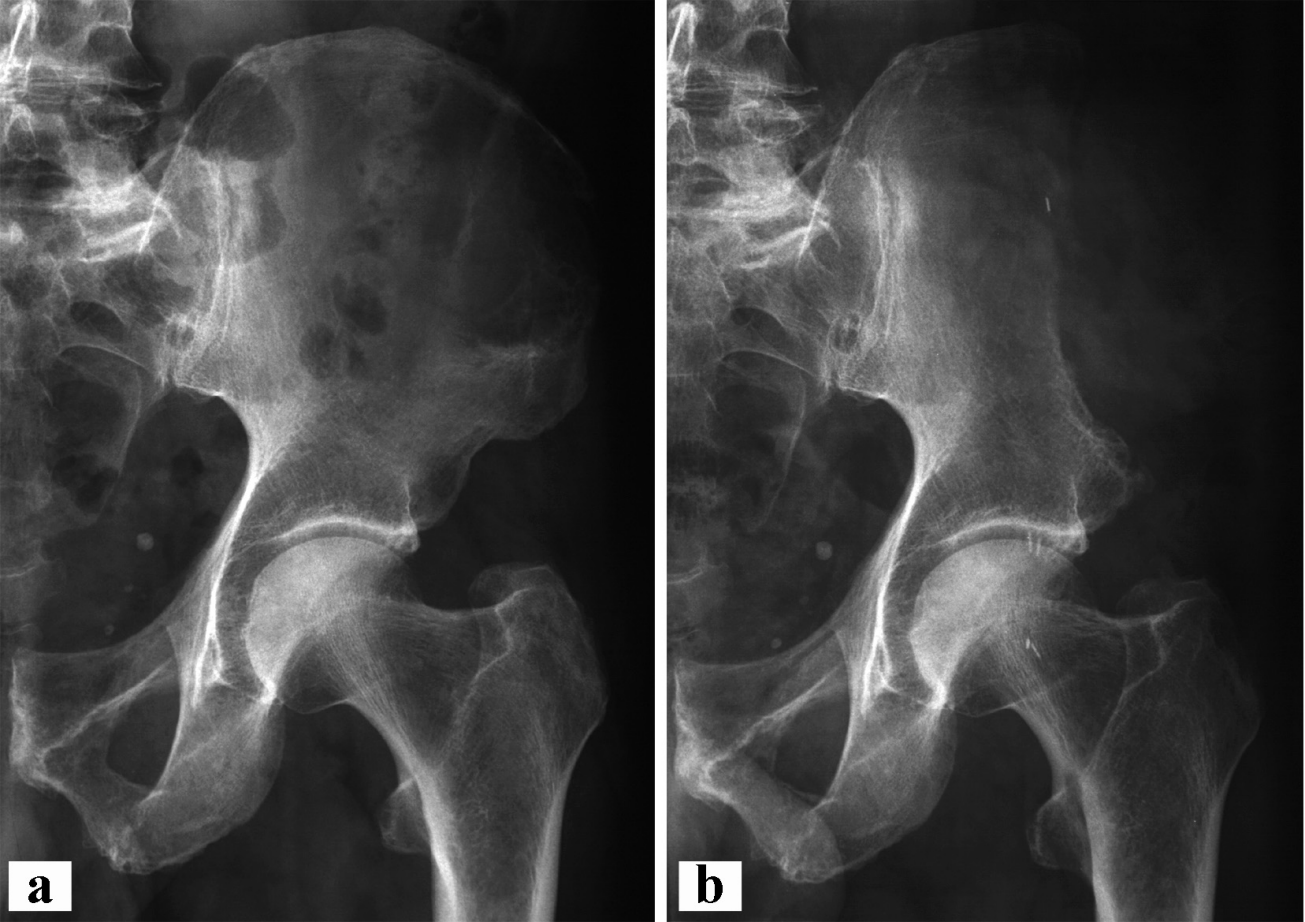
Case 7. a. Giant cell tumor of the left iliac wing. b. After wide resection of the left iliac wing without reconstruction.

The treatment regimens were divided into 5 groups. Group A included 8 patients who were treated by intralesional curettage. A small piece of cortical bone was removed to expose the lesion and the tumor was curetted through the hole using a sharp spoon, and in 3 cases by additional air drill burring. The remaining bone was left intact without destruction of the continuity of the bone. In 6 of these patients, the defect was filled with bone cement (Figure 2) and in 2 patients it was filled with bone graft. Group B included 5 patients who were treated by intralesional resection and extension of the margins by additional curettage. In contrast to intralesional curettage (group A), in these cases the bone mainly affected was resected, leading to a disruption of the bone continuity. Because the resection margins were intralesional, additional curettage (and in 3 cases, additional burring) was performed to extend the margins. In 3 of these patients, the defect was partly (Figure 3) or completely filled with bone cement. Group C included 3 patients who were treated by wide resection, all located in the ilium (Figure 4). 2 patients were treated by a combination of external beam irradiation (EBI) and surgery, and these are referred to as group D. The first patient (case 9) was initially treated by EBI as primary treatment but was referred to us after 4 months due to progression. He was treated by intralesional resection, curettage of the margins, and bone cement packing. The second (case 14) was treated by curettage and cryotherapy followed by adjuvant EBI. Group E included only 1 patient, who was solely treated by EBI with proton beam irradiation.

9 patients were also treated by selective arterial embolization (SAE) 1 day before surgery to prevent excessive bleeding. 5 of the tumors were located in region I and 4 in region II. The decision for SAE was based on proximity of the tumors to major iliac vessels seen on the magnetic resonance images.

## Results

### No major complication occurred during surgery

Most tumors with soft tissue extension were located in region I (7 of 14). In 3 patients, the information on soft tissue extension was missing (Table). In 4 cases (2, 8, 12, and 19), histology revealed a secondary aneurysmal bone cyst. 1 patient (case 19) had a soft tissue recurrence. The 1 patient who was treated solely by EBI (case 15) presented stable disease at the last follow-up, 2.5 years after EBI. All other patients had no evidence of disease at the last follow-up.

During the follow-up period no secondary sarcoma was detected, and none of the patients developed pulmonary metastases or multicentricity.

### Treatment

*Group A: intralesional curettage (8 cases).* The most common treatment regimen was group A: intralesional curettage followed by filling of the defect with bone cement (6 cases) (Figure 2). 1 of them (case 3) was referred to us due to local infection after biopsy, and was treated by curettage and bone cement packing. Another 3 local revisions and long-term antibiotics were necessary to cure the infection. 2 patients (cases 4 and 17) were referred with suspected local recurrence. In both cases, biopsy revealed scar tissue. 1 patient (case 10) presented with acetabular extension and pathological fracture of the acetabulum. He was initially treated by curettage and bone cement packing but developed femoral head necrosis with constant hip pain. An endoprosthesis with acetabular reconstruction became necessary 8 months after primary treatment. The patient was still dependent on one cane for longer walks, but was free from pain at the last follow-up.

**Figure 5.**
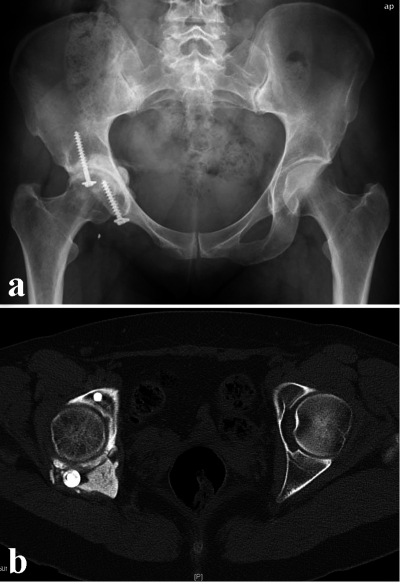
Case 11. a. After intralesional resection of a GCT of the ischium. Additional curettage and bone cement packing of the acetabular extension was performed. The cement was fixed with 2 screws to prevent dislocation. b. CT showing dislocation of the dorsal screw, necessitating surgical removal.

*Group B: intralesional resection with curettage of margins (6 cases).* 1 patient (case 12) was treated by a hip transposition (partial resection of the pelvis including the acetabulum, proximalization of the femur, and refixation of the femoral head with a Trevira attachment tube to the remaining iliac bone (Gebert et al. 2008)). Intralesional resection was performed in order to preserve the acetabulum, but was not successful due to the extension of the tumor. 14 months later, resection of painful scar tissue became necessary. At last follow-up, the patient was free from pain but was limping and dependent on a cane for longer walks, which is typical for this type of surgery (Gebert et al. 2008). In 3 patients the defect was partly (case 20, Figure 3) or completely (cases 11 and 13) filled with bone cement. In the last 2 cases, the bone cement was secured with additional screws to prevent displacement (Figure 5). 1 patient with a pubic tumor (case 19) developed local recurrence one year after surgery. She was primarily treated by intralesional resection of the pubical bone and additional curettage of the margins. The recurrence presented as a 1-cm soft tissue mass located within the scar tissue of the gluteal muscles. It was detected by routine control magnetic resonance imaging. The treatment consisted of wide resection. The patient has been free from disease for 12 months, but suffers from moderate pain in the pubic and gluteal area while sitting.

*Group C: wide resection (3 cases).* The 3 patients with tumors of region I (cases 6–8) were treated by wide resection of the iliac wing without reconstruction (Figure 4). 1 of the patients is dependent on a cane for longer walks due to limping (case 7), but the others did not show any significant impairments at the last follow-up.

*Group D: irradiation and surgery (2 cases).* The first patient (case 9) had a huge tumor of the ilium with extension to the sacrum and fifth lumbar vertebra. He had received EBI with 50 Gy for 3 months at another hospital. Due to progressive disease, he was referred to our department. We performed intralesional partial resection of the ilium and sacrum followed by bone cement packing 4 months after biopsy. Due to irradiation, wound healing was impaired by local infection—which was treated by hemipelvectomy (partial resection of the pelvis (Gebert et al. 2008)) and hip transposition 1 month later. Another month later, removal of bone cement and another debridement was performed. At last follow-up, he was dependent on a cane for longer walks and suffered from occasional local pain. The second patient (case 14) had a tumor of the pubis with acetabular extension. She was treated by intralesional curettage with additional cryotherapy, bone grafting, and capsular reconstruction with a Trevira attachment tube followed by adjuvant EBI (50 Gy for 3 months). At last follow-up after 2 years, she had no signs of disease.

*Group E: irradiation alone (1 case).* This patient (case 15) had a massive tumor of the ischiopubic area with acetabular extension and soft tissue infiltration of adductor muscles. He was treated by EBI using proton beam irradiation for 3 months. At last follow-up 2.5 years later, he presented with stable disease. Radiographs show acetabular protrusion of the femoral head, which has remained stable over the years. He also takes oral bisphosphonates as additional medication.

### Selective arterial embolization

9 patients were treated once by selective arterial embolization (SAE) the day before surgery. No complications occurred.

## Discussion

Giant cell tumors of bone rarely affect the pelvis. Only 20 (8%) of our 243 patients with GCT had a tumor of the ilium, ischium, or pubis, which is in accordance with previous reports (Campanacci et al. 1987, Sanjay et al. 1993, Balke et al. 2008b). Giant cell tumors have their peak incidence in the second and third decades of life (Campanacci 1990, Balke et al. 2008b). In a series of 19 patients with GCT of the pelvis, Sanjay et al. (1993) reported a peak age at the third and fourth decades of life (involving 13 of the 19 patients). This is supported by our findings; 15 of the 20 patients were aged between 30 and 60 years. Thus, GCT of the pelvis appears to occur in slightly older patients than the “typical” GCT of the extremities.

Almost half of the tumors (9/20) were located in the iliosacral area, which supports the findings of Sanjay et al. (1993). It must be admitted that the definition of the anatomical tumor region according to Enneking and Dunham (1978) is somewhat subjective. They divided the pelvis into 3 regions: region I, the iliosacral area; region II, the acetabular area; and region III, the ischiopubic area. In our study, 11 tumors occurred in regions II and III, and 6 tumors affected the acetabulum. They most likely originated in the ischiopubic bones and extended into the acetabular region. This is a matter of definition, because it is hardly possible to distinguish whether the tumor originated in region III and extends into region II or vice versa. Thus, a direct comparison of different studies is difficult. We can, however, conclude that tumors affecting the acetabular region are more difficult to treat. Compared to the iliac wing, wide resection is only possible with major functional impairments (case 12) and intralesional procedures often result in secondary complications such as arthritis (case 10) or problems related to cementation.

Leggon et al. (2004) indicated that the local recurrence rate for GCT of the pelvis and sacrum seems higher than for any other location. In the study by Sanjay et al. (1993), 6 of 19 pelvic tumors developed local recurrence. In our series, the recurrence rate of 1 in 20 is low. Since 3 (14) of our patients had a shorter follow-up than 2 (4) years, we cannot exclude the possibility that there might be further recurrences with longer follow-up, but 70% of local recurrences occur within the first 2 years (Balke et al. 2008a). The seemingly low recurrence rate in our patients may have been caused by several factors. 3 patients were treated by wide resection, which has been reported to prevent recurrences (Balke et al. 2008a, b), and 6 were treated by intralesional resection of the bone that was mainly affected. Thus, almost half of the patients were treated by presumably more complete tumor removal than with “typical” curettage alone, which is the recommended treatment for GCT of the extremities. Apart from this, 2 patients also received irradiation and 9 patients embolization, which might also reduce the recurrence rate. Interestingly, the only recurrence occurred within the soft tissue of the surgical tract after former intralesional resection. It would probably be more appropriate to define it as left-over soft tissue extension from the first operation rather than local recurrence. It is remarkable that such a recurrence occurs so rarely, although the surgical tract is often contaminated after intralesional procedures. We have not found any literature specifically addressing this topic.

9 of our patients were treated by SAE on the day before surgery, to prevent excessive intraoperative bleeding. The decision was made when the magnetic resonance images showed proximity of the tumor to major iliac vessels. The low recurrence rate in our series might also be—at least in part—an effect of the SAE (Misasi and Sadile 1991). In a study by Lin et al. (2002), 18 patients with GCT of the sacrum were treated with repeated intra-arterial embolization as the sole treatment. Half of the patients showed a durable radiographic response at median 9 years of follow-up. In a study by Hosalkar et al. (2007), 9 patients underwent angiography and selective arterial embolization at the time of diagnosis, followed by repeated embolization. No progression was noted in 7 of 9 cases at a mean follow-up of 9 years. It may be that presurgical selective arterial embolization not only prevents bleeding but also plays a role as an adjuvant in the treatment of large tumors.

The rate of grade III tumors (15/19) was higher than in patients with other locations (Campanacci et al. 1987, Turcotte et al. 2002, Balke et al. 2008b). Diagnosis of tumors in the pelvis may be delayed compared to tumors of the long bones. The clinical presentation is mostly uncharacteristic and may be confused with low back pain, arthritis, muscle strain, or strain of the iliosacral joint (Sanjay et al. 1993, Leggon et al. 2004). An external swelling is not visible until late, and radiographs are often misinterpreted; the osteolytic lesion of the tumor may be confused with gas-filled intestines or may be covered by shielding of ovaries or testes. This may explain the high rate of soft tissue extension (14/17). The other publications on GCT of the pelvis have not provided information about soft tissue extension (Osaka and Toriyama 1987, Sanjay et al. 1993, Leggon et al. 2004). The rate of secondary aneurysmal bone cysts in our patients seems to be relatively high (4/20). This is probably due to the size of the tumors and to the rich vascularization of the pelvis compared to the extremities.

The role of external beam irradiation (EBI) in the treatment of GCT is still a matter for discussion. It is used as primary or adjuvant treatment, but is accompanied by a high risk of inducing secondary sarcomas. The reported rates range from 7% to 25% (Johnson and Riley 1969, Sanerkin 1980, Rock et al. 1986). In the study by Sanjay et al. (1993), 8 patients with GCT of the pelvis received EBI; 2 of them developed a post-irradiation sarcoma 7 and 13 years later. In the review of the literature of pelvic and sacral GCT by Leggon et al. (2004), radiation-induced sarcoma occurred in 11% of patients who received radiation therapy for a primary or recurrent lesion at a follow-up of 5 years. The risk increases with time, so that the rate might be even higher with a longer follow-up (Tucker et al. 1987). In our study, 3 patients were treated with EBI. To date, none of the patients have developed a secondary sarcoma but the follow-up period is too short for final evaluation. We agree with Sanjay et al. (1993) that EBI is not to be recommended when an operation is a reasonable alternative. Although this is the largest published series of GCT of the pelvis, the numbers are too small for statistical analysis of different treatment regimens. However, we hope that this work will help in decision making when addressing treatment of these tumors. Because GCTs of the pelvis are extremely rare, more reliable results can probably only be obtained by using a multi-center study.
